# Indications and Limitations of vNOTES for the Surgical Staging of Early-Stage Ovarian Cancer: A Narrative Literature Review

**DOI:** 10.3390/jcm14248873

**Published:** 2025-12-15

**Authors:** Vasilios Lygizos, Dimitrios Efthymios Vlachos, Dimitrios Haidopoulos, Aikaterini Karagouni, Antonia Varthaliti, Maria Fanaki, Nikolaos Thomakos, Christos Damaskos, Nikolaos Garmpis, Gerasimos Tsourouflis, Stylianos Kykalos, Stavros Athanasiou, Dimitrios Dimitroulis

**Affiliations:** 1 1st Department of Obstetrics and Gynecology, Alexandra Hospital, National and Kapodistrian University of Athens, 115 28 Athens, Greece; vlachos.dg@gmail.com (D.E.V.); dimitrioshaidopoulos@gmail.com (D.H.); catherine.karagounis@hotmail.com (A.K.); antonia.varthaliti@hotmail.com (A.V.); maria.fanaki@gmail.com (M.F.); thomakir@hotmail.com (N.T.); stavros.athanasiou@gmail.com (S.A.); 2 Hellenic Minimally Invasive and Robotic Surgery (M.I.R.S.) Study Group, Medical School, National and Kapodistrian University of Athens, 115 28 Athens, Greece; x_damaskos@yahoo.gr (C.D.); nikosg22@hotmail.com (N.G.); gerasimos.ts@gmail.com (G.T.); kykalos@gmail.com (S.K.); dimitroulisdimitrios@yahoo.com (D.D.); 3 Department of Emergency Surgery, Laiko General Hospital, 115 27 Athens, Greece; 4 NS Christeas Laboratory of Experimental Surgery and Surgical Research, Medical School, National and Kapodistrian University of Athens, 115 28 Athens, Greece; 5 Department of Surgery, Sotiria General Hospital, 115 27 Athens, Greece; 6 Second Department of Propedeutic Surgery, Laiko General Hospital, Medical School, National and Kapodistrian University of Athens, 115 28 Athens, Greece

**Keywords:** vNOTES, ovarian cancer, minimally invasive surgery, omentectomy, hysterectomy, early-stage malignancy, feasibility, oncologic outcomes

## Abstract

Introduction: Natural Orifice Transluminal Endoscopic Surgery (NOTES) via the vagina (vNOTES) has recently appeared on the gynecology horizon as a fresh minimally invasive approach. Although vNOTES for benign adnexal conditions is being increasingly employed, very limited experiences exist for its application in ovarian tumors. In this review, the current state of vNOTES applicability for borderline ovarian tumors (BOTs) and estimated early-stage epithelial ovarian cancer (EOC) is assessed. Methods: A narrative literature review was performed to examine operative viability, perioperative safety and functional outcomes, and oncologic details as documented for patients with ovarian tumors undergoing vNOTES. Results: In the current literature, vNOTES has been utilized for adnexectomy, hysterectomy, infracolic omentectomy, peritoneal biopsies, and sampling of selective pelvic lymph nodes in carefully selected patients. The perioperative parameters—bleeding, perioperative pain, and length-of-stay indicators—have been satisfactory with minimal complications. For BOT, vNOTES can meet the requirements for all surgical goals except lymphadenectomy for metastasis evaluation for systemic management. In this context, lymphadenectomy is not necessary for BOT and therefore is no contraindication for vNOTES. However, for invasive EOC, this is a significant drawback as there is no lymphadenectomy for the evaluation and management for this complex subgroup. The oncology follow-up is prematurely limited and is heterogeneous and underpowered. Conclusions: Based on current available data, vNOTES is possible in a selected group of patients with borderline ovarian tumors and in patients with adnexal lesions that are believed to be in early-stage disease based upon imaging studies. For the treatment of invasive epithelial ovarian cancer, vNOTES should not be considered an independent staging procedure at any FIGO stage, but it might find a supplemental place in the setting of a hybrid procedure in a highly selected group of patients in an experienced center.

## 1. Introduction

Ovarian cancer is one of the leading causes of gynecologic cancer mortality amongst women because it is often diagnosed at an advanced stage. Only a small percent-approximately 20%-of ovarian cancers are detected in early stages (FIGO stage I–II), but early-stage disease carries a significantly better prognosis (5-year survival > 90% for stage I) compared to advanced-stage disease (FIGO stage III–IV) [1]. The management for presumed early-stage ovarian cancer consists of surgical staging and complete cytoreduction. The standard surgical staging includes a thorough exploration of the abdominopelvic cavity, peritoneal washings for cytology, total hysterectomy and bilateral salpingo-oophorectomy (in women who have completed childbearing), multiple peritoneal biopsies, omentectomy, resection of any visible tumor implants, and pelvic and paraaortic lymph node assessment [2]. Historically, this staging procedure has been performed via exploratory laparotomy to allow comprehensive evaluation and minimize the risk of intraoperative rupture of an ovarian malignancy. However, over the past two decades, minimally invasive surgery (MIS) techniques have been increasingly used for staging in apparent early-stage ovarian cancer. Accumulating evidence indicates that laparoscopic or robotic staging can achieve similar oncologic outcomes compared to open surgery in properly selected patients when performed by expert surgeons [3,4]. Experienced centers have reported successful laparoscopic management of early-stage ovarian carcinoma with no compromise in oncologic safety [5,6,7]. These advances in MIS have motivated the exploration of even less invasive surgical approaches for ovarian cancer.

Vaginal Natural Orifice Transluminal Endoscopic Surgery (vNOTES) is an innovative technique belonging to the MIS field that combines the advantages of endoscopic visualization with a transvaginal surgical approach. In the vNOTES procedure, the surgeon gains access to the peritoneal cavity through a colpotomy (an incision in the vaginal wall), allowing for laparoscopic instruments to be inserted via the vagina instead of transabdominal incisions. This approach eliminates visible abdominal scars and may reduce incision-related morbidity. Early clinical experience in benign gynecology has shown that vNOTES can offer several potential advantages over traditional laparoscopy, including reduced postoperative pain, shorter operative times, improved cosmetic outcomes, and faster recovery [8,9]. For example, a review published by Lerner et al. in 2023 highlighted that vNOTES hysterectomy and adnexal surgeries were associated with lower pain scores and shorter hospital stays compared to conventional laparoscopy [10]. vNOTES has rapidly gained adoption for various benign gynecologic procedures—including hysterectomy, salpingectomy, oophorectomy, ovarian cystectomy, myomectomy, and urogynecologic repairs—with high success rates and excellent patient satisfaction reported in multiple series [8]. It has also been successfully utilized in challenging patient populations, such as those with high BMI or large uteri, reinforcing its versatility [11,12].

Given the favorable outcomes of vNOTES in benign gynecology, there is increasing interest in extending this approach to oncologic surgeries. VNOTES has been used in endometrial cancer staging, where it has shown promising results. Recent studies have demonstrated that comprehensive surgical staging for early-stage endometrial carcinoma, including hysterectomy, bilateral salpingo-oophorectomy, and pelvic lymphadenectomy or sentinel lymph node, can be accomplished via vNOTES, with perioperative outcomes comparable to classic laparoscopy [13,14,15]. Wang et al. described a vNOTES hysterectomy with indocyanine green sentinel lymph node for endometrial cancer that achieved detection rates similar to the classic laparoscopic procedure [16]. A recent retrospective comparative study from Turkey reported that vNOTES staging for early-stage endometrial cancer resulted in significantly lower postoperative pain scores and reduced opioid analgesic requirements compared to laparoscopy, with no differences in operative time or short-term oncologic outcomes [13]. These findings suggest that vNOTES can be a safe and effective minimally invasive platform for gynecologic malignancies under the right circumstances.

The application of vNOTES in ovarian cancer, however, remains in its infancy. Many challenges arise in ovarian malignancies, such as the need to avoid spillage of cystic tumors to prevent upstaging, and the technical difficulty of accessing upper abdominal structures, such as the diaphragm or paraaortic lymph nodes, through the transvaginal route [17,18]. Most ovarian cancer surgeries historically require wide exposure, and even laparoscopic approaches have been adopted cautiously due to fear of missing metastatic disease or rupturing tumors. Current literature about vNOTES in ovarian cancer is limited to preliminary reports, but these early experiences are encouraging. Most published cases involving ovarian pathology have dealt with benign ovarian cysts or borderline tumors [11,19,20,21,22], with only a few studies specifically focusing on invasive ovarian cancers. An example is the preliminary study by Hurni et al., which described transvaginal omentectomy and staging in a cohort of patients with suspicious adnexal masses, demonstrating the technical feasibility of vNOTES for performing key oncologic steps like omentectomy [23]. Overall, the evidence base is poor, but it suggests that with careful patient selection and surgical expertise, vNOTES could be adopted to manage early-stage ovarian malignancies.

In this narrative literature review, we examine the feasibility and clinical application of vNOTES in patients with early-stage ovarian cancer. We review the available published case reports, case series, and comparative studies that have reported outcomes of vNOTES for ovarian malignancies, and we synthesize their findings regarding safety, efficacy, and oncologic feasibility outcomes. We also search for relevant data from vNOTES in other oncologic gynecology cancers (such as endometrial cancer) and from benign ovarian surgery, to provide context and insight into the potential advantages and limitations of this surgical approach. The goal of this review is to summarize the current state of evidence, discuss practical and oncologic considerations for using vNOTES in early-stage ovarian cancer, and outline future directions for research and clinical practice in this evolving field.

## 2. Materials and Methods

A narrative literature review was conducted to summarize the feasibility, safety, and oncologic adequacy of vNOTES in apparent early-stage ovarian cancer. PubMed, Scopus, CENTRAL, and Google Scholar were searched up to September 2025 for studies reporting vNOTES in ovarian malignancies or borderline ovarian tumors. Eligible designs included case reports, case series, and retrospective cohorts. Studies were included if they reported at least one relevant perioperative or oncologic outcome. Two reviewers screened citations and extracted data on patient characteristics, procedures performed, conversions, complications, and recurrence. Given the heterogeneity and small sample sizes, a descriptive synthesis was performed without meta-analysis.

Inclusion criteria were studies that explored vNOTES as the operative route for staging or definitive surgery in apparent early-stage ovarian malignancy/borderline tumor and reported at least one outcome listed above. Eligible designs included randomized trials (anticipated none), prospective or retrospective cohorts, comparative case–control studies, and case series with extractable quantitative data. Adult patients (≥18 years) were required. Studies enrolling mixed pathologies were eligible if ovarian cancer/borderline data were disaggregated or clearly described. Reports on vNOTES in benign adnexal disease were considered only for contextual benchmarking of perioperative metrics and not included in the core oncologic synthesis.

Exclusion criteria were studies limited to vaginal specimen extraction after conventional laparoscopy without true transvaginal endoscopic access; general NOTES or transgastric/transrectal access unrelated to gynecology; reports on non-surgical or palliative patients; case reports without outcome detail; opinion pieces, editorials, conference abstracts without full data, and unpublished theses. Studies with insufficient outcome reporting, unresolvable duplication, or critical methodological flaws (e.g., non-comparable groups with no adjustment, unclear denominators) were also excluded.

**Figure 1:**
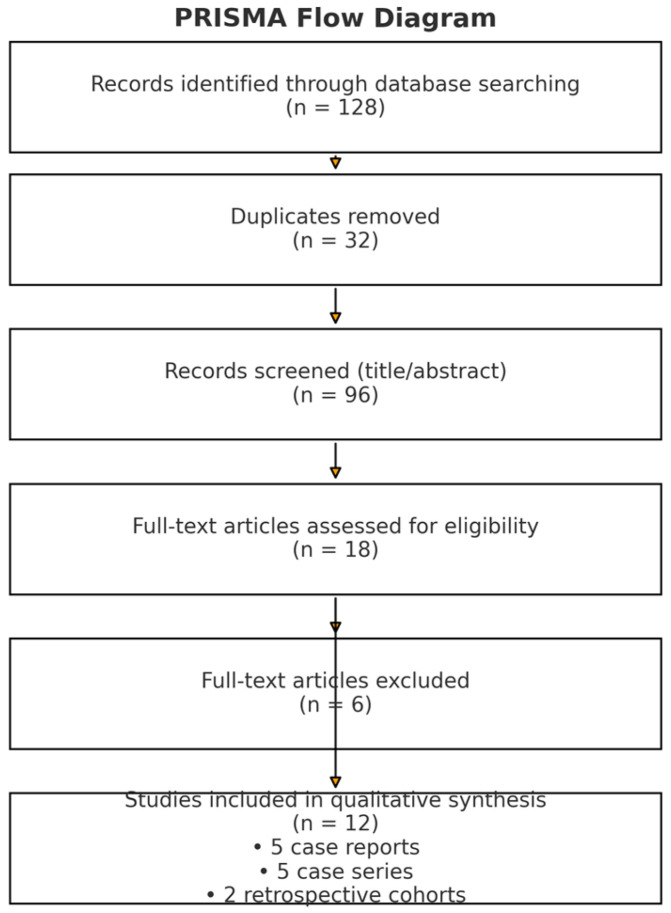
PRISMA flow diagram showing the literature identification and selection process.

We searched MEDLINE (via PubMed, 1966–2025), Scopus (2004–2025), ClinicalTrials.gov (2008–2025), Cochrane CENTRAL (1999–2025), and Google Scholar (2004–2025). The last search was performed on 12 September 2025. We also searched in reference lists of included articles and relevant reviews. The search process initially identified 128 records across all databases. After removal of 32 duplicates, a total of 96 unique citations were screened by title and abstract. Of these, 84 studies were excluded for not meeting inclusion criteria (mainly benign-only vNOTES series, non-gynecologic NOTES procedures, or insufficient data). Twelve studies were finally included in the qualitative synthesis (5 case reports, 5 case series, and 2 retrospective cohort studies). The study identification, screening, and selection process is summarized in the PRISMA flow diagram (Figure 1). Searches were limited to items published in the Latin alphabet (with pre-specified willingness to translate languages other than English, French, German, Italian, and Spanish using reliable online tools). The search strategy combined controlled vocabulary and free-text terms: “vNOTES” OR “vaginal natural orifice transluminal endoscopic surgery” OR “transvaginal NOTES” OR “natural orifice surgery “AND “ovarian cancer” OR “ovarian malignancy” OR “borderline ovarian tumor” OR “staging” OR “omentectomy” OR “peritoneal biopsy” OR “lymphadenectomy” OR “specimen containment” OR “spillage” OR “conversion” OR “length of stay”. For specificity, we did not include unrelated NOTES terms (e.g., trans gastric) and required explicit transvaginal endoscopic access in the abstract or methods (Figure 1).

Two reviewers independently screened titles/abstracts and then full texts against eligibility criteria. Disagreements were resolved by discussion. Data extracted included study characteristics (design, setting, sample size), patient selection criteria, tumor characteristics, exact vNOTES technique (access platform, ancillary ports, containment strategy), staging components performed, comparator details (when present), and all outcomes of interest. Owing to heterogeneity in design and small sample sizes, no meta-analysis was attempted; instead, we conducted a narrative synthesis emphasizing convergent signals on feasibility, safety, and early oncologic outcomes, and we highlighted areas of inconsistency and gaps to inform future research.

This approach—PICO-driven selection, multi-database retrieval to 12 September 2025, strict vNOTES definition, and combined narrative synthesis with an embedded institutional experience—was chosen to provide a focused, practice-oriented appraisal of vNOTES for early-stage ovarian cancer while transparently acknowledging heterogeneity and current evidence gaps. Study quality was assessed descriptively based on design, follow-up completeness, and clarity of denominators.

## 3. Results

### 3.1. Borderline Ovarian Tumors (BOT)

In all the included studies, the patients with borderline ovarian tumors were 22. In this subgroup, transvaginal natural orifice translumenal endoscopic surgery (vNOTES) permitted the completion of all the necessary surgical maneuvers for managing BOTs such as unilateral or bilateral adnexectomy, infracolic omentectomy, peritoneal washings, and several peritoneal biopsies. Additionally, hysterectomy was selectively carried out depending on fertility potential and other operative observations.

As the vNOTES approach lacks the necessity for lymphadenectomy for the purpose of BOT staging, the vNOTES methods were sufficient for providing the kind of staging necessary for this set of patients. As such, the lack of lymphadenectomy should not be regarded as a drawback in the context of BOT. The short-term outcomes among patients with regard to oncology were satisfactory. However, there were no recurrences documented within the short time frames allowed for the studies featured for analysis. Nonetheless, the evidence emerging from the available literature seems sufficient to conclude that vNOTES can adequately assist with borderline ovarian tumors.

The characteristics of the included studies are summarized in Table 1, detailing the design, patient numbers, histologic subtypes, procedures performed, and key outcomes relevant to early-stage ovarian malignancies.

### 3.2. Invasive Epithelial Ovarian Cancer

In all the published literature, 27 patients with allegedly early-stage invasive epithelial ovarian cancer were treated with vNOTES. In all these approaches and operations, adnexectomy was included. Additionally, hysterectomy was carried out depending on the case. The four processes were accomplished successfully without any complications. However, all the literature on vNOTES available showed that the patients underwent no systematic lymphadenectomy for the paraaortic lymph nodes. Additionally, analysis for the pelvic lymph nodes was either skipped altogether or was carried out selectively. In all these considerations and aspects for the surgical treatment and management of patients with allegedly early-stage invasive epithelial ovarian cancer and 27 with vNOTES, the complete surgical process was presumably inadequate given the criteria for surgical treatment approaches. The available oncologic outcome information for invasive disease was limited and heterogeneous. A limited number of recurrences were observed, although conclusions relative to this are highly limited by small sample size and the combination of borderline and invasive cases within several series and the limited overall duration of follow-up. Current evidence is therefore inadequate to estimate the oncologic adequacy and equivalence for laparoscopic and open staging. None of the available vNOTES series fulfilled the guideline-recommended comprehensive staging requirements for early invasive epithelial ovarian cancer, particularly regarding paraaortic lymphadenectomy. In summary, these observations demonstrate that while vNOTES is feasible for select aspects of staging surgery, the ability for vNOTES to adequately stage for invasive early-stage ovarian cancer is unproven based on consistent exclusion of paraaortic lymphadenectomy. Perioperative and early oncologic outcomes of the included studies are summarized in Table 2. Reported variables include operative time, estimated blood loss (EBL), length of hospital stay (LOS), conversion rates, complications, and follow-up duration.

Short-to medium-term oncologic follow-up (up to 36 months) demonstrated no disease-related deaths and a recurrence rate of approximately 20%, consistent with expected outcomes for early-stage epithelial ovarian carcinoma managed with conventional laparoscopy or laparotomy.

Taken together, these data demonstrate technical feasibility but highlight substantial limitations in staging completeness for invasive epithelial ovarian cancer.

## 4. Discussion

### 4.1. Early Experience with vNOTES in Ovarian Cancer Surgery

Initial studies from the beginning of the 2020s suggested that a transvaginal endoscopic method might be able to complete some tasks for adnexal and omentum surgery that were hitherto achieved by laparoscopy and laparotomy. Lowenstein et al. initially showed the potential for vNOTES infracolic omentectomy for presumed early ovarian cancer in a small clinical series with five patients and proved the ability to reach and remove the omentum with secure retrieval without conversion and significant complications [24]. It was thus shown that even non-pelvic tasks such as omentectomy can be achieved through posterior colpotomy with single-site equipment if visualization is sufficient and the disease is limited to below the upper abdomen.

Later case reports by Hurni et al. expanded the list of procedural capabilities with vNOTES for total hysterectomy, bilateral salpingo-oophorectomy, peritoneal biopsies, and infracolic omentectomy [25]. The team was able to show that complete pelvic exposure and satisfactory visualization of the lower abdomen were possible throughout the entirety of the procedure. However, while these and other cases seemed to confirm that all components of complete staging were possible with vNOTES and selected patients, the inability to accomplish paraaortic access was acknowledged to be a drawback even within these initial experiences. In any case, both publications were careful to make the point that vNOTES was limited to women with presumed stage I disease and no evidence within the upper abdomen on imaging studies.

Subsequently, Hurni and colleagues published a series with a larger number of patients and performed a vNOTES approach on a preliminary cohort of 14 patients with ovarian tumors and were able to successfully complete adnexectomy, hysterectomy, peritoneal biopsies, and omentectomy in the majority [23]. A small number underwent pelvic lymph node biopsies; however, no patients underwent systemic lymphadenectomy for the paraaortic space. Despite the excellent surgical outcomes with minimal blood loss and rapid recovery with low complication rates, such series cannot be used to determine the potential completeness for oncological staging of patients with invasive epithelial ovarian cancer.

Together, these clinical experiences with vNOTES confirm that pelvic and lower abdominal surgery may be accomplished satisfactorily on a consistent basis via the transvaginal approach for carefully selected patients. These studies form the technical basis for more comprehensive analyses and must be assessed within the context of sample size and lack of complete staging in invasive malignancies.

### 4.2. Technical Aspects and Limitations of the Transvaginal Approach

Appropriate patient selection is key to the safe application of vNOTES. Exclusion criteria that were uniformly observed across various studies include: disease limited to the pelvis on preoperative images, absence of significant upper abdominal disease that might be palpable and hinder transvaginal approach, tumor size sufficient for intact transvaginal removal, and absence of severe adhesions and recto-vaginal fibrosis that could preclude safe colpotomy [11,22,23,26,28]. Comorbidities, previous pelvic radiation, and massive endometriosis represent other potential exclusion criteria. As such, current practice for the vNOTES application for ovarian tumors is mostly for stage I disease, borderline tumors, and prophylactic adnexectomies [29,30].

Technical limitations: These relate mainly to the limited reach allowed by traditional laparoscopic instruments available from one posterior colpotomy incision. While lymph node sampling within the pelvis has been accomplished via vNOTES approaches, entry above the pelvic brim is severely limited. However, paraaortic lymphadenectomy above the inferior mesenteric artery has yet to be described within any vNOTES literature published thus far [23,26]. Should paraaortic evaluation be necessary and proven indicated, then a hybrid surgical method is necessary [26,31].

Additional ergonomic difficulties are posed by instrument overcrowding, absence of triangulation, and limited surgical angles. In benign conditions, curve analysis for learning efficiency after vNOTES suggests the operative efficiency reaches a plateau after completion of 20–30 vNOTES cases [32,33,34]. However, for cancer patients, owing to higher operative difficulty and the requirement to strictly follow the principles of tumor staging classification for proper evaluation and management, this duration would be longer. The initial practitioners would require experience and proficiency in vaginal and laparoscopic surgery along with experience with access platforms and laparoscopic instruments [9,27].

Specimen handling is another highly significant issue. In view of the potential for upgrading if malignant material spills [17,35,36], intact specimen removal is strictly necessary. It has been shown for adnexal lesions of considerable size that intact removal is feasible with colpotomy extension and impermeable containment bags [22,27,37,38,39]. Even more impressive than the rupture rate is the literature-reported minimal rate for spills: Lowenstein at al. found no ruptures among their five patients [24]; Fong et al. observed no spills [27]; and Kellerhals et al. merely one small spillage among eleven patients [26]. However, such observations must be taken with a grain of salt owing to the small series, relative excess of borderline tumors and short-term follow-up.

### 4.3. Oncologic Adequacy and the Limitations of Staging for Invasive Ovarian Cancer

The principal reason vNOTES is suboptimal for invasive epithelial ovarian cancer is the inability to offer complete staging according to current guidelines. Initial staging for apparent early-stage disease consists of pelvic and paraaortic lymph node sampling by lymphadenectomy, peritoneal washings, infracolic omentectomy, diaphragmatic evaluation, and peritoneal biopsies [37,40,41]. With all currently extant vNOTES publications to this point, there is no description of either paraaortic lymphadenectomy achieved through the transvaginal approach [23,24,25,26,27,42]. In all instances wherein lymph node sampling was accomplished, this was done selectively.

This has significant oncologic relevance. Occult lymphatic metastases can be found in as much as 15–25% of patients with putative stage I epithelial ovarian cancer [37]. Moreover, patients with bulky lymph nodes on preoperative imaging are clearly not candidates for vNOTES, while in patients without bulky nodes, comprehensive pelvic and paraaortic lymph node staging remains mandatory and cannot be achieved through a purely transvaginal approach. Inadequate sampling can result in under-staging and under-treatment, especially with more frequently nodally disseminated subtypes (e.g., serous carcinoma). Although providing good perioperative outcomes, vNOTES cannot for now be deemed a suitable independent approach for complete staging for invasive ovarian cancer.

In the malignant series described by Fong et al., recurrence occurrences were noticed even with uneventful perioperative courses; however, the small number and variability in stage make survival analysis impossible [27]. The available length of follow-up is limited to a mean of 12–36 months and is hardly sufficient to determine recurrence rates on a long-term scale. Also included were patients with borderline lesions and non-epithelial malignancies [11,23,26].

Despite being increasingly utilized for endometrial cancer [11], SLN mapping is still investigational for ovarian cancer and has yet to be published for vNOTES-based SLN studies. Until now, there has been a lack of effective methods for exploration of the upper retroperitoneum, and in the absence of validated SLN-based strategies, vNOTES should be considered suboptimal by itself in the staging procedure of invasive epithelial ovarian cancers of any FIGO staging.

### 4.4. Comparison with Conventional Minimally Invasive Approaches

Although operative time and recovery parameters for vNOTES and adnexal vNOTES procedures appear to be superior for selected benign and adnexal vNOTES cases [11,22,43,44,45], the current evidence cannot be extrapolated to support vNOTES as similar to multiport laparoscopy and robotic surgery for ovarian cancer with respect to oncology equivalence. Traditional approaches for minimally invasive surgery allow for pelvic and paraaortic lymphadenectomy and evaluation for diaphragm and upper abdomen biopsies—steps critical for staging [6,18,37]. Thus, while vNOTES may offer cosmetic and clinical advantages for patients, such advantages do not take precedence over the absolute requirements for operative staging for invasive cancer.

New robotic approaches for transvaginal surgery, such as transvaginal single-port vNOTES (SP-vNOTES), may potentially extend the surgical field for transvaginal surgery [46,47,48]. However, these methods are still under investigation and have yet to be assessed with respect to ovarian cancer.

### 4.5. Future Directions and Research Priorities

However, several gaps exist within the evidence. Studies thus far have been retrospective with small sample sizes and included various indications. To the best knowledge, there are no multicenter prospective and randomized studies examining vNOTES for ovarian cancer. The mean duration of surveillance within the available literature is short and rarely exceeds 36 months. This is inadequate given that ovarian cancer recurrence exceeds three years [18,25,49].

Future priorities include multicenter prospective registries with standardized reporting for operative procedures, extent of staging, and recurrence rates [26], uniform definitions for hybrid methods and indications for conversion, systemic reporting of disease-free survival (DFS), overall survival (OS), and recurrence patterns. Another priority is the assessment of cost-effectiveness, sexual function, and quality of life [8,9,11]. Technological advancements such as fluorescence-guided surgery [16], enhanced access platforms, and robots may potentially increase the scope of staging. Training programs and simulator models [50,51] would be required for safe transfer and dissemination of the method.

Until then, vNOTES may be regarded as a potential minimally invasive tool for the management of borderline ovarian tumors and selected adnexal pathologies, while its application for invasive epithelial ovarian cancer is still exploratory at this stage [52].

## 5. Conclusions

Based on the current state of available evidence, the Natural Orifice Translumenal Endoscopic Surgery (NOTES) in the vaginal approach (vNOTES) is an evolving “incisionless” minimal access platform that aims to leverage the strengths of traditional vaginal surgery while incorporating laparoscopic views of the surgical space. Initial experience in benign gynecology has shown less postoperative pain, quicker mobilization, and excellent cosmetic results. Based upon the success achieved in benign gynecology, a steadily increasing number of studies have explored the application of vNOTES in adnexal masses, borderline ovarian cancers, and putative early ovarian cancers. Taken in the aggregate, the available literature has shown that vNOTES has enabled the accomplishment of adnexectomy, hysterectomy, infracolic omentectomy, peritoneal washings, and biopsy through the vaginal approach in a totally “incisionless” manner. Despite the absence of a routine lymphadenectomy in borderline tumors, the available studies suggest a safety profile for the achievement of the required surgical goals in vNOTES procedures with little bleeding, low complications, and a short in-hospital stay ranging from 1 to 3 days without any unexpected safety signals, including those of port-site metastases, and a low rate of intraoperative rupture.

The oncologic principles have been maintained with the reception of the entire specimen in protective bags and the absence of morcellation. The question of oncologic equivalence should be made with caution, owing to the current studies involving a small subset of patients and a significant number of borderline and non-epithelial lesions with less follow-up. Secondly, the important limitation regarding vNOTES is its inefficiency in performing the complete staging in the case of invasive epithelial ovarian cancers. Neither pelvic nor paraaortic lymphadenectomy has been demonstrated systematically by the transvaginal approach alone, in a way that the studies available either do not include the nodal staging or include it in the form of a selective pelvic biopsy.

The current application of the technique appears to be best suited in the context of borderline cancers and risk-reducing surgeries, where the need for staging is low and the cosmetic advantage of scarless and minimally invasive surgery is highly sought after. Future studies include enhanced training programs, technological development, robotic SP-vNOTES, and the study of strategies involving the sentinel node. Until multicenter studies with long-term follow-up are performed, vNOTES should be considered an investigational procedure and be restricted to selected patients in highly experienced centers.

## Figures and Tables

**Table 1:** Characteristics of included studies on vNOTES in early-stage ovarian cancer and borderline ovarian tumors *(Abbreviations: OC = ovarian cancer; BOT = borderline ovarian tumor; BSO = bilateral salpingo-oophorectomy; TH = total hysterectomy; USO = unilateral salpingo-oophorectomy; LND = lymphadenectomy; IDS = interval debulking surgery; NACT = neoadjuvant chemotherapy)*.

Author (Year)	Study Type	Country/Institution	No. of Patients	Histology	FIGO Stage	Procedures Performed	Conversion	Follow-Up (Months)	Key Findings/Notes
Lowenstein et al. (2020) [24]	Case series	Israel	5	Suspicious/early epithelial OC	I–II	Omentectomy	0	24	Feasible omentectomy via vNOTES; minimal blood loss.
Hurni et al. (2022) [25]	Case reports	Switzerland	2	1 LGSC/1 benign cystadenofibroma	I	Hysterectomy + BSO + omentectomy ± biopsies	0	18	Complete staging achieved; no complications.
Hurni et al. (2023) [23]	Case series	Switzerland	14	Early epithelial OC/BOT	I	TH + BSO + omentectomy ± biopsies	0	30	All completed via vNOTES; 1 minor infection.
Kellerhals et al. (2025 [26]	Case series	Switzerland	11	7 OC + 4 BOT	I	TH/USO ± BSO + omentectomy ± LND	0	12	One cyst rupture; otherwise uncomplicated.
Fong et al. (2025) [27]	Retrospective cohort	Singapore	19	Malignant OC (12 staging, 4 fertility-sparing, 3 IDS)	I–II/post-NACT	BSO/TH ± omentectomy ± LND	0	26	4 recurrences (21%), no deaths; feasibility confirmed.
Other minor series/reports		Various	<5 each	BOT/low-grade OC	I	vNOTES BSO ± omentectomy	0	≤12	Feasibility demonstrated; short follow-up.

**Table 2:** Perioperative and early oncologic outcomes of vNOTES for early-stage ovarian cancer and borderline ovarian tumors across published studies.

Author (Year)	No. of Patients (Malignant + BOT)	Operative Time (Min)	EBL (mL)	LOS (Days)	Conversion (%)	Complications (%)	Spillage (%)	Follow-Up (Months)	Recurrence (%)	Key Notes
Lowenstein 2020 [24]	5 (3 + 2)	115 ± 25	50 ± 20	1.0	0%	0%	0%	24	0%	Complete staging feasible; no cyst rupture.
Hurni 2022 [25]	2 (1 + 1)	130	80	1	0%	0%	0%	18	0%	Hybrid vNOTES + laparoscopy case.
Hurni 2023 [23]	14 (9 + 5)	135 (90–180)	60 (30–120)	1.5 (1–2)	0%	7% (minor)	0%	30	7% (1 recurrence)	Reproducible complete staging.
Kellerhals 2025 [26]	11 (7 + 4)	120 ± 20	70 ± 40	1.3 ± 0.5	0%	0%	9% (1 minor)	12	0%	One minor spillage; no conversions.
Fong 2025 [27]	19 (19 + 0)	142 ± 34	75 ± 40	1.8 ± 0.7	0%	5%	0%	26 (12–48)	21% (4 recurrences)	Largest malignant-only cohort; includes primary staging, fertility-sparing malignant cases, restaging, and interval debulking.
Others (<5 each)	≈5 (3 + 2)	100 (80–130)	50 (20–80)	1	0%	0%	0%	≤12	0%	Feasibility shown in isolated cases.

## Data Availability

No new data were created or analyzed in this study. Data sharing is not applicable.
